# Global Use of Casein Glycomacropeptide Protein Substitutes for Phenylketonuria (PKU): Health Professional Perspectives

**DOI:** 10.3390/nu18030488

**Published:** 2026-02-02

**Authors:** Sharon Evans, Rani Singh, Kirsten Ahring, Catherine Ashmore, Anne Daly, Suzanne Ford, Maria Ines Gama, Maria Giżewska, Melanie Hill, Fatma Ilgaz, Richard Jackson, Camille Newby, Alex Pinto, Martina Tosi, Ozlem Yilmaz Nas, Juri Zuvadelli, Anita MacDonald

**Affiliations:** 1 Birmingham Children’s Hospital, Steelhouse Lane, Birmingham B4 6NH, UK; catherine.ashmore@nhs.net (C.A.); a.daly3@nhs.net (A.D.); maria.gama1@nhs.net (M.I.G.); alex.pinto@nhs.net (A.P.); anita.macdonald@nhs.net (A.M.); 2 Department of Human Genetics, Emory University School of Medicine, Atlanta, GA 30322, USA; rsingh@emory.edu; 3 The PKU Clinic, Kennedy Centre, Centre for Paediatric and Adolescent Medicine, Copenhagen University Hospital, Rigshospitalet, 2100 Copenhagen, Denmark; kirsten.ahring@regionh.dk; 4 National Society for Phenylketonuria (NSPKU), Sheffield S12 9ET, UK; suzanne.ford@nspku.org; 5 Department of Pediatrics, Rare Diseases and Metabolic Medicine, Pomeranian Medical University in Szczecin, 71-252 Szczecin, Poland; maria.gizewska@pum.edu.pl; 6 Sheffield Teaching Hospitals NHS Foundation Trust, Sheffield S5 7AU, UK; melanie.hill13@nhs.net; 7 Department of Nutrition and Dietetics, Faculty of Health Sciences, Hacettepe University, Ankara 06100, Turkey; fatma.celik@hacettepe.edu.tr; 8 Department of Health Data Science, University of Liverpool, Liverpool L69 3GJ, UK; richj23@liverpool.ac.uk; 9 Bristol Royal Hospital for Children, Upper Maudlin St, Bristol BS2 8BJ, UK; camille.newby@uhbw.nhs.uk; 10 Department of Health Sciences, University of Milan, 20142 Milan, Italy; martina.tosi@unimi.it; 11 Department of Nutrition and Dietetics, Ankara University, Ankara 06760, Turkey; ozlmylmz102@gmail.com; 12 Clinical Department of Pediatrics, San Paolo Hospital, ASST Santi Paolo e Carlo, 20142 Milan, Italy; juri.zuvadelli@asst-santipaolocarlo.it

**Keywords:** casein glycomacropeptide, phenylketonuria, PKU, protein substitute, dietitian, physician, questionnaire

## Abstract

Background/Objectives: Casein glycomacropeptide (cGMP) has been modified to enable its suitability as a low phenylalanine (Phe) protein substitute (PS) in phenylketonuria (PKU). No data is available about its global usage. Methods: A 60-item multiple choice and short answer/extended response questionnaire examining the use of modified cGMP in PKU was distributed globally to dietitians and physicians via web-based professional inherited metabolic disorder groups. Results: Respondents (*n* = 208) from 45 countries across 6 continents completed the questionnaire. Of these, 83.7% (*n* = 174) were dietitians/nutritionists, 14.9% (*n* = 31) medical doctors/physicians and 1.4% (*n* = 3) other health professionals, caring for both paediatric and adult patients (59.1%), paediatrics only (25.0%) or adults only (15.9%). cGMP PS were reported as not available in their centre/hospital by 19.7% (*n* = 41), mostly in Africa, South America, and southern and western Asia. The main reasons included lack of regulatory approval (65.8%), not promoted by manufacturers (41.5%), and cost (29.3%). An estimated 25% of represented patients globally were using cGMP PS; 78.4% (*n* = 163) following refusal/poor adherence with Phe-free amino acids and 54.8% (*n* = 114) for adult patients recommencing dietary treatment. There were concerns about the residual Phe in cGMP negatively impacting blood Phe levels in children <12y (66.3%), adolescents (48.0%), adults (34.6%), and the first trimester of pregnancy (53.1%). Sixty nine percent (*n* = 145) adjusted dietary Phe prescription according to the cGMP Phe content, particularly in regions with a higher percentage of severe PKU variants. Commonly perceived clinical advantages with cGMP were improved taste/palatability (93.2%, *n* = 194) and fewer gastrointestinal symptoms (55.8%, *n* = 116). Perceived clinical disadvantages were residual Phe (72.1%, *n* = 150), lack of data in children < 3 years (48.1%, *n* = 100), and the high energy content of some brands (45.2%, *n* = 94). There were concerns that cGMP PS were too high in sugar (34.1%, *n* = 71) and dissatisfaction or uncertainty about the adequacy of its Phe (66.3%) and amino acid (34.1%) content. Conclusions: There is global inconsistency in access to cGMP PS suitable for PKU, and in the interpretation of evidence-based research. Some professionals have significant concerns about its nutritional composition particularly residual Phe, limiting its estimated use to approximately 25% of PKU patients globally.

## 1. Introduction

Phenylketonuria (PKU) is a rare autosomal recessive disorder of amino acid metabolism that necessitates lifelong dietary management to prevent irreversible neurocognitive impairment [1]. Central to treatment is the restriction of the amino acid phenylalanine (Phe) and the provision of low-Phe protein substitutes. Traditionally, these protein substitutes consist of synthetic amino acids, which, despite their efficacy, are often associated with poor palatability and suboptimal adherence [2,3,4,5].

Casein glycomacropeptide (cGMP), a bioactive peptide derived from cheese whey, is an alternative protein source for individuals with PKU [6]. Unlike amino acid-based formulas, cGMP may offer improved taste and texture, which may enhance dietary adherence [6,7,8]. Preliminary studies suggest that cGMP-containing protein substitutes confer additional physiological benefits, including improved nitrogen retention [9,10], reduced blood Phe variability [11], enhanced body composition [12] and potential prebiotic, antimicrobial, and immunomodulatory effects [13,14].

However, cGMP is not Phe-free. Its residual Phe content poses a particular challenge for individuals with classical PKU and low Phe tolerance, especially when cGMP is used as the sole protein substitute source [15,16,17]. Moreover, while some clinical benefits have been observed, many (such as gut microbiota enhancement) are extrapolated from non-PKU populations or animal models [13,18], and long-term data in human PKU cohorts remain limited. Currently there is insufficient clinical evidence to suggest any beneficial impact on growth, body composition, nutritional biochemistry, kinetics, blood Phe variability, microbiota, bone or satiety [9,11,12,14,16,18,19,20,21]. Interpretation of studies on cGMP is complicated by the considerable heterogeneity among the different cGMP protein substitute products (powder, liquid, bars) in terms of their energy, amino acid and overall nutritional content [22]. Study results are also affected by patient Phe tolerance, their baseline blood Phe levels, and the amount of protein equivalent provided by cGMP (33% to 100% of protein substitute intake) [23]. Therefore, important questions remain regarding their optimal amino acid composition, age-specific suitability, and comparative efficacy across PKU phenotypes.

Despite over 15 years of experience with cGMP in PKU, their clinical application and perceived value among healthcare professionals are unclear. The international availability of cGMP products, national approval and funding mechanisms, and the extent to which dietitians recommend cGMP as a first-line protein substitute, particularly across different age groups, remain poorly documented. Furthermore, the influence of regional marketing strategies may have shaped professional attitudes and prescribing behaviours, yet this has not been systematically explored.

As cGMP-based protein substitutes become increasingly integrated into PKU dietary management, understanding global health professionals’ perspectives is essential to inform future product development, guide clinical research priorities, and support evidence-based policy decisions. Accordingly, the aim of this study was to design and implement an international survey to characterise the global use of cGMP-based protein substitutes among health professionals managing individuals with PKU. Specifically, the survey sought to: (1) document patterns of cGMP availability, procurement pathways and use across regions; (2) explore professional perceptions of its clinical benefits and limitations; and (3) assess attitudes regarding its suitability across different age groups and life-stage categories.

In this manuscript, the term protein substitute refers to products classified as medical foods in the United States and as medical formulas or special medical foods in other regions.

## 2. Materials and Methods

### 2.1. Questionnaire Development

A 60-item, non-validated questionnaire (Supplementary Material, Figure S1) was developed using Microsoft Forms (Microsoft Corporation, Redmond, WA, USA) and distributed to health professionals involved in the care of individuals with PKU. The purpose of the survey was to examine international clinical use and professional perceptions of cGMP-based protein substitutes. The questionnaire comprised multiple-choice, Likert-scale, short-answer, and extended-response items. Initial questions assessed whether respondents had access to cGMP-based protein substitutes and, where access was lacking, sought to identify the specific barriers. For respondents with access, the survey explored patterns of clinical use, including preferred patient age groups and prescribing practices.

Additional domains explored information on national approval and funding mechanisms for protein substitutes, procurement pathways for cGMP protein substitutes, comparative cost considerations relative to amino acid-based protein substitutes, and the range of cGMP formulations available. The questionnaire also assessed professional satisfaction with current cGMP-based products, focusing on nutritional composition, product format, and marketing, as well as perceptions of their clinical advantages and limitations.

### 2.2. Pilot Study

Following development of the questionnaire, expert opinion was obtained from 9 inherited metabolic disorder (IMD) dietitians from 3 countries to assess content validity. The experts evaluated each item in terms of language, clarity and relevance of the construct being measured. Revisions were made as required.

### 2.3. International Distribution

The questionnaire was distributed internationally to health professionals involved in the care of patients with IMD (specifically PKU), including those that did not have access to cGMP. This was conducted by advertising through health professional groups across the continents of Europe, North America, South America, Australasia, Africa and Asia, including: the Society for the Study of Inborn Errors of Metabolism (SSIEM); the British Inherited Metabolic Disease Group (BIMDG); the Genetic Nutrition Online Metabolic Listserv (GNO-METAB-L) which is hosted by the Emory Medical Nutrition Therapy for Prevention (MNT4P) programme in collaboration with the Genetic Metabolic Dietitians International (GMDI) organisation; the Australasian Society for Inborn Errors of Metabolism (ASIEM); the Japanese Society for Inherited Metabolic Diseases (JSIMD); the Chinese Society for Metabolic Biology (CSMB); the Indian Society of Inborn Errors of Metabolism (ISIEM); the Latin American Society of Inborn Errors of Metabolism and Newborn Screening (SLEIMPN); the Society for Inherited Metabolic Disorders (SIMD) and North American Metabolic Academy (NAMA). The questionnaire was open between November 2024 and May 2025. Regular reminder emails were sent to ensure a broad range of responses internationally. It was anticipated that a total of 150 responses (pragmatically aiming for a minimum of 5 from each continent) would be sufficient to be representative, including at least 10 from countries with no access to GMP-containing protein substitutes. However, the absence of baseline data on the size and composition of the IMD workforce in each region prevented determination of what would constitute a truly representative global sample.

### 2.4. Data Analysis and Statistics

Returned questionnaires were then collated and analysed statistically. Quantitative data are summarised as frequencies of counts with associated percentages. Analyses are presented as tabulated summaries and bar plots to demonstrate the responses between continents. The key aim was to explore any difference in response between continents. Qualitative data from open comments were grouped into themes and tabulated.

### 2.5. Ethical Approval

Ethical approval was not required as there was no patient involvement, no change in patient treatment, and no collection of respondents’ personal or identifiable data. Ethical review and approval were not required according to the NHS Health Research Authority and Medical Research Council “Is my study research” online decision tool [24] (Supplementary Material, Table S1).

## 3. Results

### 3.1. Study Group

A total of 208 health professionals from 45 countries across 6 continents completed the survey (Table 1), collectively representing the care of approximately 14,200 individuals with PKU. The majority of respondents were dietitians (*n* = 174; 84%), with physicians comprising 15% (*n* = 31) of the sample and other health professionals comprising 1% (*n* = 3). Most respondents (*n* = 123; 59%) reported caring for both paediatric and adult patients with PKU, while 25% (*n* = 52) worked exclusively with paediatric populations and 16% (*n* = 33) with adults only.

Nearly half (*n* = 102; 49%) of the health professionals were employed full-time in IMD, 34% (*n* = 70) worked at least half-time, and 17% (*n* = 36) were engaged less than half-time in this speciality. Patient caseloads varied, but 61% of respondents (*n* = 127) reported managing 50 or more individuals with PKU (Table 2).

In most cases, the decision regarding the type of protein substitute to prescribe was made by the dietitian or nutritionist (58%, *n* = 120), or jointly between the dietitian/nutritionist and the patient or caregiver (54%, *n* = 112). Fewer respondents reported that prescribing decisions were made by physicians alone (21%, *n* = 44), jointly between the physician and patient/caregiver (20%, *n* = 41), by the patient/caregiver independently (16%, *n*= 33), or by a multidisciplinary team (11%, *n* = 22) (Note: respondents were able to select more than one response).

### 3.2. Access to cGMP-Based Protein Substitutes

Access to cGMP-based protein substitutes varied significantly across regions, with the lowest levels of approval (based on those who responded) in Asia (24/28), South America (5/8), Eastern Europe (4/14) and Africa (*n* = 1/1) (Table 1).

Eighty-two percent (*n* = 170) of respondents reported that cGMP-based protein substitutes were approved for use in their country or region and 1% (*n* = 2) were in the process of being approved. Fourteen percent (*n* = 29) reported that they were not approved for use in their country or region, with an additional 3% (*n* = 7) unsure of their approval status.

**Figure 1:**
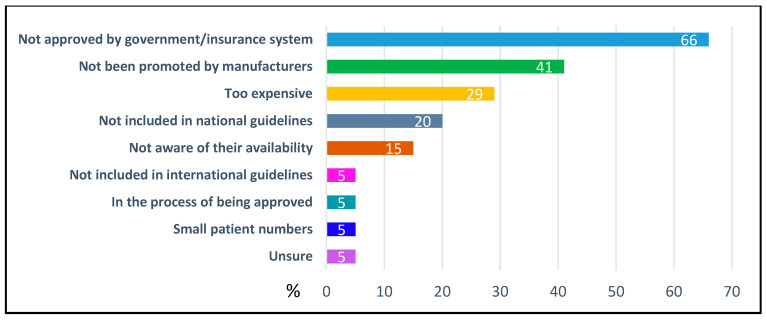
Reasons for lack of access to cGMP-based protein substitutes (% of respondents; *n* = 44).

Irrespective of national approval status, 79% (*n* = 164) of respondents indicated that cGMP-based protein substitutes were available for patient use, 20% (*n* = 41) said they were not available and 1% (*n* = 3) were unsure. The most cited barrier was lack of government or insurance system approval (66%, *n* = 27/41) (Figure 1). Other reasons included limited patient numbers and restricted hospital supply due to the infrequency of admissions. These access limitations were not confined to any specific country, region, or continent (Figure 1).

#### 3.2.1. Regulatory Approvals and Payment Processes for Protein Substitutes

Seventy-nine percent of respondents (*n* = 165) reported that new protein substitutes required national or regional approval before they could be prescribed to patients. Despite this regulatory hurdle, 76% (*n* = 158) indicated that protein substitutes were fully subsidised by government or insurance systems. In contrast, 6% (*n* = 12) stated that patients were required to pay the full cost of all protein substitute with these responses primarily from Hong Kong, Singapore, India, and parts of Australia and the United States. Twenty-one percent (*n* = 44) reported partial subsidy, meaning patients contributed to the cost; and 15% (*n* = 32) reported that full coverage was available only under specific circumstances, such as for children, individuals in full-time education, or those who were unemployed, with this model most commonly reported in England, Tunisia, Cyprus, and parts of the USA.

Several centres described mixed funding arrangements. For example, in the USA, Medicaid (a state-funded programme for low-income individuals and families) provided full coverage in some states, while private insurance coverage varied widely. As a result, patients within the same centre could experience full, partial, or no subsidy depending on their insurance status.

#### 3.2.2. Local Restrictions on Use of cGMP Protein Substitutes

Among respondents with access to cGMP-based protein substitutes (*n* = 164), 60% (*n* = 99/164) reported that a minimum age for use was specified in their country or region. This was primarily attributed to the range and branding of available products. An additional 18% (*n* = 29/164) were unsure if a minimum age limit applied.

Of those who reported a defined age threshold (*n* = 99), the most cited minimum age was 3 years (46%, *n* = 46), followed by 4 years (21%, *n* = 21), 1 year (16%, *n* = 16), and 3–4 years (6%, *n* = 6) (Table 3). Notably, 5% (*n* = 5) indicated that cGMP-based protein substitutes had been used in children younger than the stated minimum age when clinically justified.

Fifteen percent of respondents (*n* = 32/208) reported that cGMP-based protein substitutes were not approved for use during pregnancy in their country or region. These responses were received mostly from countries/regions where cGMP was not routinely available (Brazil, Chile, Estonia, Hong Kong, Mexico, Tunisia, Turkey), but also from Belgium, Hungary, Italy, Latvia, Poland, Spain, and the United States. An additional 25% (*n* = 53) were unsure of the approval status for use in pregnancy.

#### 3.2.3. Cost of cGMP-Based Protein Substitutes and Issues with Supply

Thirty percent of respondents (*n* = 62/208) reported that cGMP-based protein substitutes were more expensive than amino acid-based alternatives. However, 51% (*n* = 106) were either unsure or did not have access to cGMP products, and 19% (*n* = 40) stated that cGMP products were not more expensive. Reported costs varied both within and between countries, with South and North America (62% and 50%) and Eastern Europe (50%) reporting cGMP being more expensive and Oceania reporting not (57%).

Among respondents actively using cGMP-based protein substitutes (*n* = 164), 45% (*n* = 74) had experienced supply interruptions, and 9% (*n* = 15) were unsure if supply issues had occurred (Table 4). Supply disruptions varied regionally, most frequently being reported in North America (76%), Oceania (67%) and Northern Europe (58%), particularly in geographically isolated regions such as New Zealand. Reasons for supply interruption were multifactorial but the majority (64%, *n* = 47/74) were attributed to manufacturing or supplier-related issues.

### 3.3. Clinical Practice with cGMP-Based Protein Substitutes

#### 3.3.1. Number of Patients Prescribed cGMP-Based Protein Substitutes

Among the estimated 14,200 individuals with PKU represented by survey respondents reporting their patient numbers, approximately 3850 (25%) were currently prescribed cGMP-based protein substitutes.

Twenty-eight percent of respondents indicated that up to 25% of their patients were prescribed cGMP, 24% suggested usage in up to 50% of their patient cohort, and 6% in up to 75% (Table 5).

Higher uptake, defined as use in up to 50% or up to 75% of patients, varied regionally, most reported in North America, Oceania, and Southern Europe. Countries with the highest reported usage included New Zealand, the United States, Canada, Portugal, Germany, and Italy, where at least half of respondents indicated that cGMP products were prescribed to 50–75% of their patients. In contrast, respondents from Asian and African countries reported no use of CGMP-based protein substitutes.

#### 3.3.2. Clinical Advantages and Disadvantages as Perceived by Respondents

**Figure 2:**
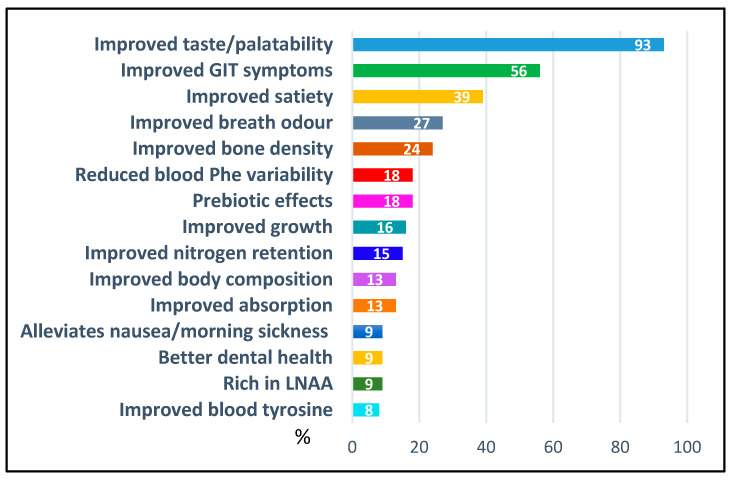
Perceived clinical advantages of cGMP-based protein substitutes (% of respondents, *n* = 208). Abbreviations: GIT = gastrointestinal, Phe = phenylalanine, LNAA = large neutral amino acids.

**Figure 3:**
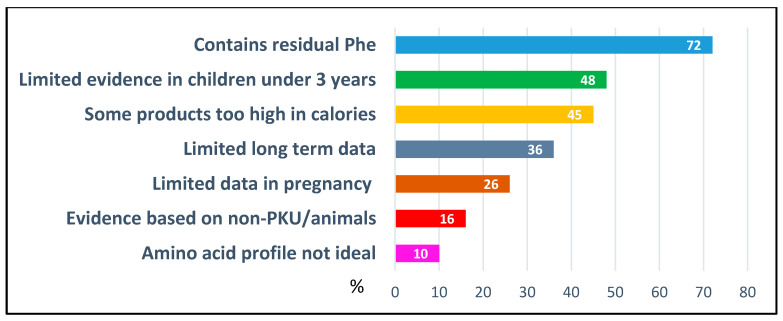
Perceived clinical disadvantages of cGMP-based protein substitutes (% of respondents, *n* = 208). Abbreviations: Phe = phenylalanine; PKU = Phenylketonuria.

Respondents were asked to identify perceived clinical advantages and disadvantages of cGMP-based protein substitutes compared with traditional amino acid-based formulations (Figure 2 and Figure 3). Improved palatability (93%, *n* = 194/208) and gastrointestinal tolerance (56%, *n* = 116) were most frequently cited perceived advantages, whereas residual Phe (72%, *n* = 150) and limited evidence supporting use in children under 3 years of age (48%, *n* = 100) remained major perceived disadvantages.

Perceived advantages of cGMP-based protein substitutes varied by geographic region. Improved satiety was cited more frequently in North and South America (75%) than in other regions (0–57%). Improved bone density was more commonly reported in South America (50%), Asia (43%), and Southern Europe (42%) compared to other areas (0–25%). Perceived body composition benefits were highest in Eastern Europe (29%), Southern Europe (21%), South America (25%), and Asia (21%), with lower endorsement elsewhere (0–9%). Improved absorption of cGMP was noted by 22% of respondents in North America, versus 0–15% in other regions. Overall, respondents from Asia and South America demonstrated the greatest divergence in perceived clinical benefits, with consistently higher or lower endorsement rates across multiple domains.

Perceived disadvantages of cGMP-based protein substitutes were largely consistent across regions. The most notable variation concerned energy content, which was identified as a concern by 100% of respondents in Oceania and 73% in Northern Europe, compared to 0–55% in other regions.

#### 3.3.3. Clinical Scenarios Where cGMP-Based Protein Substitutes Are Used as a First Choice

Respondents identified several key clinical scenarios in which cGMP-based protein substitutes would be preferentially used. The most common reasons were poor adherence to amino acid-based protein substitutes (78%, *n* = 163/208) and outright refusal to take them (78%, *n* = 162). cGMP was also frequently recommended for patients recommencing dietary treatment in adulthood (55%, *n* = 114).

Additional indications included use in patients with poor metabolic control (32%, *n* = 66), pregnant women experiencing nausea and vomiting with amino acid-based substitutes (28%, *n* = 58), and individuals receiving adjunct pharmacological treatment such as sapropterin (19%, *n* = 40).

#### 3.3.4. Recommended Number of Daily Doses cGMP

The most recommended dosing frequency across all age groups for cGMP-based protein substitutes was three times per day (49%, *n* = 81/164). However, dosing practices varied, with many respondents indicating that frequency was tailored to individual factors such as patient preference (55%, *n* = 90/164) or blood Phe levels (37%, *n* = 61/164).

#### 3.3.5. Concerns About Residual Phe in cGMP-Based Protein Substitutes

**Figure 4:**
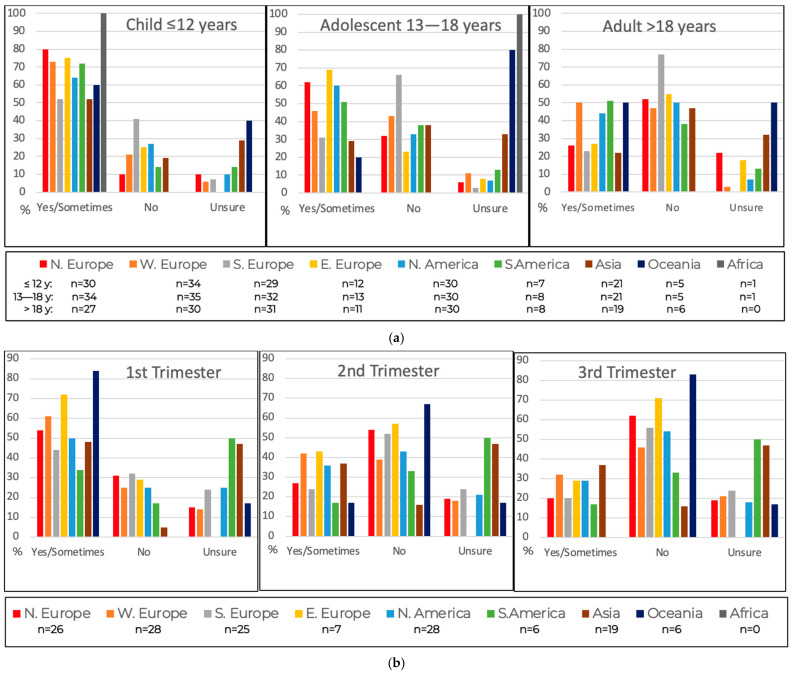
Does residual phenylalanine in cGMP-based protein substitutes negatively affect blood phenylalanine (**a**) in different age groups? (**b**) during pregnancy? Note: not all respondents cared for patients from all age groups or maternal PKU patients.

Sixty-seven percent (*n* = 112/169) of respondents caring for children reported that they considered the residual Phe content in cGMP-based protein substitutes negatively affected blood Phe levels in children under 12 years of age (Figure 4a). Concern decreased with age: 48% (*n* = 86/179) of those caring for adolescents expressed concern for teenagers, and 35% (*n* = 56/162) for adults. Amongst respondents involved in maternal PKU, 53% (*n* = 77/145) were concerned about the residual Phe in cGMP for women in the first trimester of pregnancy (Figure 4b). This declined to 32% (*n* = 47/145) in the second trimester and 26% (*n* = 38/145) in the third trimester. An additional 11% to 24% of respondents were uncertain about the impact of residual Phe across these age groups and life stages.

Concern about the impact of residual Phe in cGMP varied significantly by region. Respondents from Eastern and Northern Europe expressed the highest levels of concern for children and teenagers while those from Southern Europe and Asia were least concerned. Western Europe, South America and Oceania were the most concerned for adults, with Southern Europe least concerned. Oceania and Eastern Europe reported the greatest concern regarding cGMP use during the first trimester of pregnancy, with a progressive decline in concern across the second and third trimesters. Respondents from Asia and Oceania demonstrated the highest levels of uncertainty about the effects of residual Phe on blood Phe across all age groups and life stages.

When asked if respondents would lower the dietary Phe prescription according to the Phe content of the cGMP-based protein substitute, 21% (*n* = 43/208) said yes and 49% (*n* = 102/208) said sometimes (Table 6). Those most likely to change the prescription varied regionally with Eastern and Northern Europe and Oceania more likely to change than those from Southern Europe.

### 3.4. Satisfaction with the Nutritional Profile of cGMP-Based Protein Substitutes

#### 3.4.1. Phe Content of cGMP-Based Protein Substitutes

Differences in opinion regarding the Phe content of cGMP-based protein substitutes were common among respondents. Of 208 surveyed professionals, 31% (*n* = 64) considered the Phe content too high, 34% (*n* = 70) judged it appropriate, and 36% (*n* = 74) were unsure (Table 7). Regional variation was evident: concerns about excessive Phe were most frequently reported in Northern Europe, while respondents from Southern Europe predominantly viewed the content as appropriate. Uncertainty was highest among respondents from Asia, Oceania, Africa, and Eastern Europe.

#### 3.4.2. Tyrosine and Overall Amino Acid Content

Respondents expressed mixed views about the tyrosine content of cGMP-based protein substitutes. Forty-two percent (*n* = 88/208) considered the tyrosine content adequate, 4% (*n* = 9) felt it was too low, 1% (*n* = 2) too high, and 52% (*n* = 109) were unsure.

Overall satisfaction with the amino acid profile of cGMP products was relatively high, with 66% (*n* = 137) reporting satisfaction. However, 31% (*n* = 65) expressed uncertainty, and a small proportion (3%, *n* = 6) reported dissatisfaction.

Generally, free-text responses highlighted widespread unease about the amino acid composition of cGMP-based protein substitutes. Concerns were raised about amino acid bioavailability, particularly the kinetics of single amino acids added to cGMP and their interaction within blended formulations. Some noted that patients changing entirely to cGMP may receive fewer amino acids overall, potentially affecting nutritional adequacy.

#### 3.4.3. Energy Content

Almost one quarter of respondents (24%, *n* = 49/208) expressed concern about the overall energy content of cGMP-based protein substitutes, while a further 25% (*n* = 52) were unsure. There was considerable regional variation in response, with dissatisfaction most reported by respondents from Southern (45%, *n* = 15/33) and Northern Europe (35%, *n* = 16/45) and Oceania (29%, *n* = 2/7), whereas those from North America, Eastern, and Western Europe were generally more satisfied (58–81% satisfied).

Conversely, in free text comments some respondents from the USA called for higher-energy options to better support younger children and toddlers, particularly those requiring a higher protein-equivalent intake. Others advocated for a broader range of energy profiles to accommodate diverse patient needs, particularly in pregnancy. Respondents from the UK and Germany emphasised the importance of offering cGMP products with a varied energy content to support personalised dietary management.

**Figure 5:**
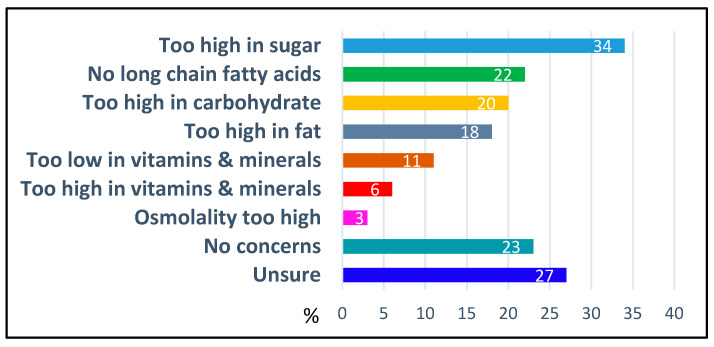
Concerns about the nutritional quality of cGMP-based protein substitutes (% of respondents, *n* = 208).

Thirty-four percent (*n* = 71/208) of respondents were concerned that the sugar content of cGMP products was too high and 20% (*n* = 42) had concerns about the carbohydrate content, particularly for adult and overweight patients. A further 27% (*n* = 56) were unsure about the overall nutritional quality (Figure 5).

Comments from Australia, Italy, England, and Wales highlighted the need for reduced-energy or “lite” alternatives, especially for bars and ready-to-drink formats. Respondents noted that a high energy content could deter health professionals from prescribing cGMP and recommended re-evaluating the carbohydrate and fat composition of some brands to improve suitability.

#### 3.4.4. Vitamin and Mineral Content

Concerns about the vitamin and mineral composition of cGMP-based protein substitutes were reported by respondents from 20 countries and were not confined to any specific region. The most frequently cited micronutrients of concern included calcium (9%; *n* = 18), vitamin D (9%; *n* = 18), iron (5%; *n* = 11), selenium (5%; *n* = 10), vitamin B12 (4%; *n* = 8), vitamin A (3%; *n* = 7), zinc (3%; *n* = 6), and omega-3 fatty acids (3%; *n* = 6). Free-text responses reinforced these concerns. Respondents from Canada, the USA, Argentina and Greece reported that calcium, iron, magnesium, sodium, and potassium could exceed tolerable upper intake levels in adults with high protein requirements. Vitamin D was also flagged as excessive in some formulations (Norway, USA, Canada), and high levels of vitamin A were reported to be of concern in the UK and Portugal. Excessive vitamin A was highlighted as a concern particularly for pregnant women (Spain, UK). Others suggested that omega-3 and -6 fatty acids were consistently low across many products (Australia, Poland, UK). Respondents from the Netherlands and France noted that some products lacked adequate micronutrients for children under 10 years of age (particularly biotin, vitamin B12, iron, potassium, zinc and selenium) and Germany noted low selenium levels in routine blood tests. Micronutrient content was noted to vary between product brands and certain nutrients were insufficient for younger age groups but exceeded recommended upper levels in older patients. Several respondents advocated there was a need for tailored formulations, particularly in maternal PKU, with adjusted iron, folic acid, and vitamin A content (Greece), and a simplified approach to micronutrient dosing (UK).

While most respondents (67%, *n* = 140/208) supported the inclusion of vitamins and minerals in cGMP-based protein substitutes, 17% (*n* = 36) disagreed and 15% (*n* = 32) were unsure. Regional variations were apparent; those who disagreed were predominantly from Oceania (57%, *n* = 4/7), Northern Europe (29%, *n* = 13/45), and North America (28%, *n* = 9/32). The primary reasons cited were concerns about excessive micronutrient intake in individuals with higher protein requirements (*n* = 27), such as pregnant women, adolescents engaged in bodybuilding, adults with elevated needs, or those using multiple protein substitutes. Additional concerns included the impact of fortification on taste (*n* = 11), and the need for flexibility in product choice, with 19 respondents advocating for both fortified and unfortified options.

Free-text comments from New Zealand, Australia, and Norway recommended offering vitamin- and mineral-free formulations to allow for additional protein equivalent without surpassing micronutrient upper safe levels of intake. Taste was also a recurring theme, with some suggesting that separating micronutrient supplementation from protein substitutes could improve palatability and adherence (Italy, Norway, Sweden, UK).

### 3.5. Presentation and Preferences of cGMP-Based Protein Substitutes

**Figure 6:**
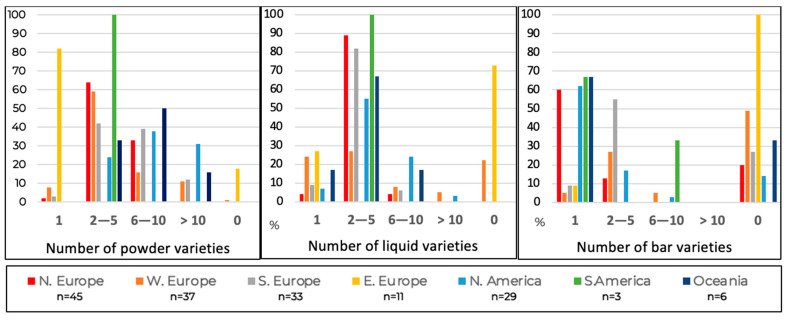
Presentation of cGMP protein substitutes available (% of respondents).

Of those using cGMP products (*n* = 164), most centres reported access to between two and five different cGMP-based protein substitutes in liquid (62%, *n* = 101/164) or powdered (47%, *n* = 77/164) formats. A smaller proportion had access to six or more varieties (11%, *n* = 18 liquid; 40%, *n* = 66 powdered) (Figure 6). In contrast, cGMP protein substitute bars were less widely available: 32% (*n* = 53) of centres had no bar products, 34% (*n* = 56) had only one variety, and 26% (*n* = 43) offered two or more.

Regional disparities were evident. Centres in North America, Northern and Southern Europe, and Oceania reported the greatest diversity of cGMP products across all formats. In comparison, centres in Asia and Africa were least likely to have access to cGMP products of any type.

There was no strong consensus among respondents regarding preferred formats of cGMP-based protein substitutes. Forty percent of respondents (*n* = 84/208) reported no preference, while 45% (*n* = 94) favoured liquid formulations, 38% (*n* = 79) powdered products, and 15% (*n* = 32) selected bars as their preferred format. Powdered products were most popular among respondents from North and South America and Northern Europe, while liquid formulations were similarly favoured in North and South America and Southern Europe. cGMP bars were most preferred in Eastern Europe and Asia. Respondents from Africa and Oceania predominantly reported no specific format preference.

Among respondents with access to cGMP-based protein substitutes, 74% (*n* = 121/164) reported satisfaction with the available flavour options. Suggestions for improvement included broader presentation formats, such as powder sachets and smaller-volume liquids, as well as expanded flavour profiles. Respondents requested savoury options, adult-oriented flavours (e.g., coffee and lemon), and dairy-inspired varieties such as cheese and yoghurt.

**Figure 7:**
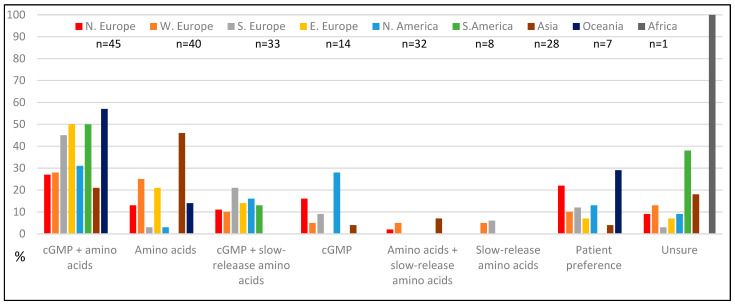
Clinical preference for protein substitutes for PKU (% of respondents, *n* = 208).

When asked about their clinical preference for protein substitutes in PKU management, over half (55%, *n* = 115/208) favoured cGMP products either alone or in combination with amino acid-based substitutes (Figure 7). This combined approach was the most preferred across all continents except Asia, where amino acid-based products were favoured. North America showed the strongest preference for cGMP alone (28%, *n* = 9/32). Thirteen percent of respondents (*n* = 26), primarily from Oceania and Northern Europe, expressed no specific clinical preference stating that it was dependent on individual patients and their preference.

The primary reasons cited by respondents for selecting a protein substitute centred on accessibility, clinical experience, patient preference, adherence with taking protein substitute, blood Phe control, and perceived clinical advantages.

## 4. Discussion

This study represents the first global survey to examine health professionals’ perspectives on the use of cGMP-based protein substitutes in PKU. Findings demonstrate substantial variation in cGMP use worldwide, shaped by national regulatory systems, product availability, and clinician familiarity.

Although cGMP-based substitutes have been available internationally for more than 15 years, their adoption among healthcare professionals remains limited, with marked regional differences. Current estimates based on represented regions indicate that only around 25% of the global PKU population uses cGMP products. Uptake has been influenced not only by regulatory approval pathways and market access but also by clinical attitudes that guide prescribing practices. Health professionals mostly prescribed cGMP in situations of poor adherence or refusal of amino acid-based substitutes, and for adults returning to dietary treatment. Its use during pregnancy, in individuals with suboptimal metabolic control, and alongside adjunct pharmacotherapies such as sapropterin reflects growing recognition of cGMP’s potential role in challenging or transitional care scenarios. However, the absence of robust safety and efficacy data in children under three years of age and during pregnancy continues to limit broader adoption.

Access to cGMP-based protein substitutes remained restricted across several regions, including much of Asia, South America, Eastern Europe, and Africa. Beyond limited availability, the diversity of product formats (powders, liquids, and bars) varies both within and between countries. Product preference appeared to correlate with clinical experience and availability, particularly in regions with constrained access. Geographical isolation was associated with more frequent supply interruptions, further challenging consistent and equitable access.

There were international differences in clinical guidance regarding cGMP-based protein substitutes for PKU. Notably, differences between North American and European approaches reflect varying interpretations of the available evidence and regulatory priorities [25]. The 2014 guidelines from the American College of Medical Genetics and Genomics (ACMG) offered limited commentary on cGMP, stating only that “the availability of a variety of medical food products gives patients and their providers many options to facilitate compliance with dietary Phe restriction,” while cautioning that “medical food choices may also impact nutritional status, reinforcing the need for careful monitoring” [26]. The updated 2023 ACMG evidence-based guidelines did not expand on this position, providing no further recommendations regarding cGMP use [1]. In contrast, the recent revised European guidelines [23] suggest that based on current evidence “cGMP can be prescribed as a protein substitute in patients aged ≥4 years provided that its Phe content is considered in the daily Phe allocation and that the profile of all amino acids meets age-appropriate requirements”. The strength of the recommendation was marked as “strong” and the level of evidence “moderate,” with 75% of experts agreeing, and 20% mostly or partially agreeing.

Findings from our international survey demonstrated substantial heterogeneity in clinical opinion regarding the use of cGMP-based protein substitutes, including notable variation within individual countries. This variability likely reflects differences in clinical experience, product availability, the absence of definitive evidence, and divergent interpretations of the scientific literature. Improved palatability, enhanced gastrointestinal tolerance, and increased satiety were the most frequently reported advantages of cGMP-based substitutes [6,7,9,17,20,27,28,29,30,31], although evidence supporting effects on satiety remains inconsistent.

Concerns about residual Phe content remained prominent, particularly for children under 12 years of age and during early pregnancy. They were most frequently reported by health professionals in Eastern and Northern Europe and Oceania, regions where dietary Phe prescriptions are more commonly adjusted to account for the Phe contribution from cGMP products and where a substantial proportion of paediatric cGMP research on Phe tolerance with cGMP has been undertaken [15,16,17]. Patients in these regions also tend to present with more classical PKU [32,33], resulting in lower daily Phe tolerance and heightened sensitivity to even small variations in Phe intake. This is particularly a concern in women with classical PKU in the early stages of pregnancy, when even small increases in blood Phe may pose risks to fetal development [34].

Geographic differences in clinical opinion may reflect regional disparities in research activity, dissemination practices, and commercial promotion. For example, improved satiety is more frequently cited as a benefit in North and South America, where much of the supporting evidence has originated from American research centres [7,31]. In contrast, studies from Northern Europe [17,19,20] and Southern Europe [35] have not demonstrated superior satiety outcomes with cGMP compared to amino acid-based protein substitutes and findings from non-PKU populations have further questioned any satiety advantage [36,37,38]. Similarly, improved absorption and nitrogen retention are more commonly perceived as benefits in North America, likely influenced by regional studies reporting favourable outcomes [9,10]. Conversely, European studies have not identified significant differences in absorption between cGMP and amino acid-based formulations [19,39].

These discrepancies in study results may be partly attributable to the substantial heterogeneity in cGMP product composition across studies, particularly in the inclusion and concentration of large neutral amino acids (LNAAs) [22]. In addition, protein equivalent provided by cGMP and baseline Phe concentrations and Phe tolerance/consumption metrics vary across studies [40]. Establishing consistent terminology may help to improve global clarity.

Concerns regarding nutritional composition focused primarily on energy density and sugar content, particularly for adults and individuals with overweight or obesity. cGMP-based protein substitutes generally contain higher levels of sugars and saturated fats than amino acid-based products, especially in ready-to-drink formats and snack bars [22]. Energy-density concerns were reported most frequently in Northern and Southern Europe and Oceania, and least often in North America and Asia. This pattern may reflect regional research priorities: European and Oceanic studies have more extensively examined weight status and body composition in PKU [22,41,42,43,44,45,46,47,48,49,50,51,52,53], whereas the North American and Asian evidence base remains comparatively limited [54].

Micronutrient composition was also highlighted as a concern in cGMP-based protein substitutes, with some health professionals suggesting that current formulations may not fully meet age-specific or life-stage nutritional requirements. Some advocated for the availability of both fortified and unfortified products to support flexible prescribing and patient-centred care. Evidence on micronutrient status in patients using cGMP protein substitutes remains limited, but studies have reported no significant differences compared with amino acid-based products [21,55]. One study observed higher plasma vitamin D and folate concentrations in cGMP users [14], although sample sizes were small.

Although no clinical studies have evaluated the growth and safety of cGMP use in children under 4 years of age [23], it was given to children under 3 years in Canada, Denmark, Germany, Poland, Spain, and the USA. In addition, some cGMP formulations may not provide an amino acid profile that meets World Health Organization (WHO) recommendations for young children, raising concerns about nutritional adequacy [23].

This study has several limitations. Despite being “globally” disseminated, this study depended on voluntary involvement through professional web-based forums, which resulted in uneven regional representation and self-selection bias. The uneven distribution of respondents, especially the underrepresentation in areas with the least access, may overstate or underestimate actual worldwide patterns. While a pragmatic minimum of five responses per continent was anticipated, only one response was received from Africa, and the majority originated from Europe and North America. Consequently, all analyses were necessarily exploratory and descriptive. The absence of baseline data on the size and composition of the IMD workforce in each region prevented determination of what would constitute a truly representative global sample. The geographical imbalance may also reflect selection bias toward English-speaking respondents, limited product availability across much of Asia and South America, and smaller population sizes in Oceania.

Despite wide dissemination through IMD speciality networks, uptake from non-dietetic professionals remained limited. This is likely to have influenced the perspectives captured and limited the extent to which the findings represent the full multidisciplinary care team. Overall, these findings reflect health professionals’ perceptions and self-reported practice rather than objectively verified clinical behaviour. As such, responses may be shaped by individual experience, local practice norms, and personal interpretation of the evidence base. The use of a non-validated questionnaire is an additional limitation. Formal psychometric validation was not feasible given the global scope, linguistic diversity, and exploratory nature of the study; however, the absence of validation introduces uncertainty regarding construct validity, reliability, and cross-cultural comparability. The questionnaire was also subject to recall bias, and respondents varied in their familiarity and experience with cGMP products, which may have influenced the accuracy and consistency of responses.

Differences in global terminology (e.g., “medical food” vs. “medical formula”) and variation in health-care systems, prescribing practices, and product availability may have contributed to misinterpretation of specific items. Furthermore, the cross-sectional design captures perceptions at a single point in time and cannot account for evolving clinical practice, emerging evidence, or changes in product availability. Finally, global usage estimates are based solely on regions that responded to the questionnaire.

Despite these limitations, there is growing clinical interest in cGMP-based options for younger children. This is driven by the challenges associated with transitioning older patients who have been accustomed to amino acid-based protein substitutes from infancy. The absence of age-appropriate cGMP formulations and supporting safety data represents a critical gap in the therapeutic landscape for PKU and highlights the need for targeted research in early childhood populations.

## 5. Conclusions

In summary, global variation in the adoption of cGMP in PKU is shaped by numerous factors. National regulatory frameworks, reimbursement structures, and historical reliance on amino acid-based formulas all influence clinical uptake. Professional attitudes toward the nutritional adequacy, its Phe content, safety, and therapeutic role of cGMP also vary, contributing to divergent practices and interpretations of emerging evidence. Greater harmonisation of terminology and clearer guidance on the appropriate use of cGMP are needed. International collaboration among professional societies and regulatory agencies will be essential to ensure equitable access to cGMP and other novel protein substitutes. To support more consistent and equitable integration of cGMP into PKU management, stronger international alignment in clinical guidance, supported by robust scientific evidence, is warranted.

## Figures and Tables

**Table 1:** Continents and countries/regions represented by respondents.

Continent	Countries/Regions	% (*n*)	% (*n*) of Individual Respondents Reporting That cGMP Is Not Approved/Unsure in Their Country/Region
Europe—Northern	Denmark, England, Finland, Iceland, Ireland, Northern Ireland, Norway, Sweden, Wales	22 (45)	0 (0)
Europe—Western	Austria, **Belgium**, France, Germany, Netherlands, Switzerland	19 (40)	3 (1/40)
Europe—Southern	Greece, Italy, Portugal, Spain	16 (33)	0 (0)
Europe—Eastern	**Estonia**, **Georgia**, Hungary, Latvia, **Moldova**, Poland, Romania, **Slovenia**	7 (14)	29 (4/14)
North America	Canada, **Mexico**, USA	15 (32)	3 (1/32)
South America	Argentina, **Brazil**, **Chile**	4 (8)	63 (5/8)
Asia	**Cyprus**, **Hong Kong**, **Iran**, India, **Malaysia**, Saudi Arabia, **Singapore**, **Taiwan**, **Turkey**	13 (28)	86 (24/28)
Oceania	Australia, New Zealand	3 (7)	0 (0)
Africa	**Tunisia**	1 (1)	100 (1/1)
TOTAL		100 (208)	17 (36/208)

**Bold:** Countries/regions where cGMP was not approved for use in PKU in at least some centres or there was uncertainty about approval.

**Table 2:** Number of patients with PKU cared for by respondents.

Number of Patients	% (*n*) of Respondents
<10	7 (14)
10–20	10 (21)
21–50	22 (46)
51–100	23 (48)
101–150	15 (32)
>150	23 (47)

**Table 3:** Minimum age for use of cGMP-based protein substitutes.

Minimum Age	Countries	% (*n*) Respondents *n* = 99
<1 year	India, USA	2 (2)
1 year	Canada, Denmark, Germany, Spain, USA	16 (16)
2 years	Germany, Poland	2 (2)
1–3 years	USA	1 (1)
3 years	Australia, Belgium, Denmark, France, Germany, Greece, Hungary, Italy, Latvia, Netherlands, Norway, Portugal, Spain, Sweden, Switzerland, UK, USA	46 (46)
4 years	Austria, Belgium, France, Netherlands, Poland, UK	21 (21)
3–4 years	Australia, France, Italy, Portugal, UK	6 (6)
6 years	Slovenia	1 (1)
11 years	Italy (adult only centre)	1 (1)
As per manufacturer’s instructions	Spain, UK, USA	3 (3)

**Table 4:** Percentage of respondents from each country experiencing interruption to supplies of cGMP protein substitutes.

Continent	Country	% (*n*) of Respondents with Interrupted Supply/Total Number with Access	Total % (*n*) by Region
	Denmark	50 (2/4)	
	England	58 (14/25)	
	Finland	0 (*/1)	
	Iceland	0 (0/1)	
Northern Europe	Ireland	0 (0/1)	
	Northern Ireland	100 (4/4)	58 (26/45)
	Norway	50 (1/2)	
	Sweden	80 (4/5)	
	Wales	50 (1/2)	
	Austria	0 (0/2)	
	Belgium	0 (*/6)	
Western Europe	France	63 (5/7)	16 (6/37)
	Germany	9 (1/11)	
	Netherlands	0 (*/9)	
	Switzerland	0 (*/2)	
	Greece	100 (1/1)	
Southern Europe	Italy	25 (4/16)	36 (12/33)
	Portugal	20 (1/5)	
	Spain	55 (6/11)	
	Latvia	50 (1/2)	
	Moldova	0 (0/1)	
Eastern Europe	Poland	33 (2/6)	27 (3/11)
	Romania	0 (*/1)	
	Slovenia	0 (*/1)	
North America	Canada	67 (4/6)	76 (22/29)
	USA	78 (18/23)	
South America	Argentina	33 (1/3)	33 (1/3)
Oceania	Australia	50 (2/4)	67 (4/6)
	New Zealand	100 (2/2)	
TOTAL			45 (74/164 **)

* Some respondents were unsure if patients had experienced interruptions in supply of cGMP products. ** *n* = 41 reported cGMP was not available; *n* = 3 were unsure if available.

**Table 5:** Percentage of patients by continent currently prescribed at least some cGMP-based protein substitutes.

Continent	% (*n*) of Patients Taking Some cGMP Protein Substitutes						
	Up to 75%	Up to 50%	Up to 25%	Up to 10%	Up to 5%	None	Total *n*
North America	16 (5)	50 (16)	28 (9)	3 (1)	3 (1)	0 (0)	32
Oceania	29 (2)	29 (2)	0 (0)	29 (2)	14 (1)	0 (0)	7
Southern Europe	9 (3)	33 (11)	36 (12)	12 (4)	9 (3)	0 (0)	33
Northern Europe	2 (1)	24 (11)	44 (20)	20 (9)	4 (2)	4 (2)	45
Western Europe	3 (1)	20 (8)	30 (12)	18 (7)	20 (8)	10 (4)	40
Eastern Europe	0 (0)	7 (1)	21 (3)	14 (2)	21 (3)	36 (5)	14
South America	0 (0)	0 (0)	25 (2)	0 (0)	13 (1)	63 (5)	8
Asia	0 (0)	0 (0)	0 (0)	0 (0)	0 (0)	100 (28)	28
Africa	0 (0)	0 (0)	0 (0)	0 (0)	0 (0)	100 (1)	1
TOTAL	6 (12)	24 (49)	28 (58)	12 (25)	9 (19)	22 (45)	208

**Table 6:** Percentage of respondents who would adjust and lower the dietary Phe prescription according to the Phe content of cGMP products.

	Yes % (*n*)	Sometimes % (*n*)	No % (*n*)	Total *n*
Northern Europe	24 (11)	58 (26)	18 (8)	45
Western Europe	20 (8)	45 (18)	35 (14)	40
Southern Europe	6 (2)	36 (12)	58 (19)	33
Eastern Europe	50 (7)	36 (5)	14 (2)	14
North America	3 (1)	69 (22)	28 (9)	32
South America	38 (3)	13 (1)	50 (4)	8
Asia	36 (10)	43 (12)	21 (6)	28
Oceania	14 (1)	86 (6)	0 (0)	7
Africa	0 (0)	0 (0)	100 (1)	1
TOTAL	21 (43)	49 (102)	30 (63)	208

**Table 7:** Satisfaction with the Phe content of cGMP products (number of respondents).

	Too High % (*n*)	Unsure % (*n*)	Just Right % (*n*)	Total *n*
Northern Europe	53 (24)	24 (11)	22 (10)	45
Western Europe	38 (15)	23 (9)	40 (16)	40
Southern Europe	24 (8)	15 (5)	61 (20)	33
Eastern Europe	21 (3)	64 (9)	14 (2)	14
North America	38 (12)	22 (7)	41 (13)	32
South America	13 (1)	37 (3)	50 (4)	8
Asia	0 (0)	86 (24)	14 (4)	28
Oceania	14 (1)	71 (5)	14 (1)	7
Africa	0 (0)	100 (1)	0 (0)	1
TOTAL	31 (64)	36 (74)	34 (70)	208

## Data Availability

The raw data supporting the conclusions of this article will be made available by the authors on request.

## Supplementary Materials

The following supporting information can be downloaded at: https://www.mdpi.com/article/10.3390/nu18030488/s1, Figure S1: Questionnaire; Table S1: “Is my study research” online decision tool.

## Abbreviations

The following abbreviations are used in this manuscript:

ACMG	American College of Medical Genetics and Genomics
ASIEM	Australasian Society for Inborn Errors of Metabolism
BIMDG	British Inherited Metabolic Diseases Group
cGMP	casein glycomacropeptide
CSMB	Chinese Society for Metabolic Biology
GMDI	Genetic Metabolic Dietitians International
IMD	Inherited Metabolic Disease
ISIEM	Indian Society of Inborn Errors of Metabolism
JSIMD	Japanese Society for Inherited Metabolic Diseases
KSIMD	Korean Society of Inherited Metabolic Disease
NAMA	North American Metabolic Academy
Phe	phenylalanine
PKU	phenylketonuria
LNAA	Large neutral amino acids
SIMD	Society for Inherited Metabolic Disorders
SIMMESN	Dietetics Working Group of our Italian Society for Metabolic Diseases
SLEIMPN	Latin American Society of Inborn Errors of Metabolism and Newborn Screening
SSIEM	Society for the Study of Inborn Errors of Metabolism
TYR	Tyrosinaemia
