# Supplementary material for: Global Use of Casein Glycomacropeptide Protein Substitutes for Phenylketonuria (PKU): Health Professional Perspectives
Source: Nutrients. 2026 Feb 2;18(3):488. doi: 10.3390/nu18030488 (PMC12899376; doi:10.3390/nu18030488)
Supplement: Supplementary file 1 [file nutrients-18-00488-s001.zip › Figure S1.pdf]

A survey to understand current practices in the global use of CGMP for PKU, TYR & ALK

A survey to understand current practices in the global use of Casein Glycomacropeptide (CGMP) for Phenylketonuria, Tyrosinaemia and Alkaptonuria

Introduction
This questionnaire has been developed by dietitians in the UK (Birmingham Children's Hospital) and USA (Emory University School of Medicine) to examine the global use of modified casein glycomacropeptide (CGMP) in inherited metabolic disorders. Over the last 15 years, CGMP has been used as a low phenylalanine and low tyrosine protein substitute (medical food) for phenylketonuria (PKU), tyrosinaemia (TYR) and alkaptonuria (ALK). CGMP based protein substitutes contain some residual phenylalanine or tyrosine. There is no data available about their global usage, how they are used in clinical practice and any barriers that countries or individual dietitians may have associated with their use. We would like to explore these areas in more depth.

We would like to invite you to complete this questionnaire. More than one person from your centre/department can respond. The more information we have from all of you, explaining your practice, the more we will understand about the use of CGMP protein substitutes. There are separate questionnaires for each condition (PKU, TYR and ALK), each one should take approximately 15-20 minutes to complete. For some questions you have the option to select more than one response.

When we have a representative global sample of questionnaires, we will collate the responses and a paper will be prepared for publication in a peer reviewed journal. All contributors will be acknowledged at the end of the publication if they choose to leave their name. The published study results will be used to improve the evidence base for CGMP products for PKU, TYR and ALK.

DEMOGRAPHIC SCREENING QUESTIONS

1. Are you a: *

- ◯ Dietitian/nutritionist
- ◯ Diet assistant
- ◯ Nurse
- ◯ Medical doctor/physician
- ◯ Yes
- ◯ Other

2. If you wish to be acknowledged as a contributor on the final published paper for this survey please enter your full name below. If you wish to remain anonymous please leave this blank.

3. Which hospital/centre do you work at? Please give the **name of your hospital/centre**, **department**, **city/state** and **country**. *

4. What percentage of your working hours are dedicated to inherited metabolic disorders? *

- 100%
- 50-99%
- <50%

PHENYLKETONURIA (PKU)

5. Do you care for patients with **phenylketonuria (PKU)** on dietary treatment? *

- ◯ Yes
- ◯ No

6. Do you normally care for paediatric or adult patients with **PKU**, or both? *

- ◯ Paediatric (0-18 years)
- ◯ Adult (>18 years)
- ◯ Both paediatric and adult

7. Approximately how many patients with **PKU** on dietary treatment do you care for (including those on drug treatment e.g. sapropterin, but excluding those with hyperphenylalaninaemia on a normal diet and patients treated with sapropterin or Palynziq who are on a normal diet i.e. on no protein substitute/medical food)? *

- ◯ <10
- ◯ 10-20
- ◯ 21-50
- ◯ 51-100
- ◯ 101-150
- ◯ >150

Access to CGMP based protein substitutes/medical foods

8. Who is responsible for choosing the type of protein substitute/medical foods that you use in your hospital/centre for **PKU (tick all that apply)**? *

- [ ] Dietitian/nutritionist
- [ ] Doctor/physician
- [ ] Multidisciplinary team
- [ ] Patients/carers
- [ ] Joint decision between patient/carer and dietitian/nutritionist
- [ ] Joint decision between patient/carer and doctor/physician
- [ ] Other

9. Are CGMP based protein substitutes/medical foods approved for use in **PKU** by regulatory processes in your country/region/state? *

- Yes
- No
- They are in the process of being approved
- Unsure

10. Do you have any comments about the previous question?

11. Is there a minimum age for use of CGMP based protein substitutes/medical foods for use in **PKU** in your country/region? *

- Yes
- No
- Unsure
- CGMP not available

12. If yes, from what age can CGMP based protein substitutes be used for **PKU**? *

13. Are CGMP based protein substitutes/medical foods approved for use in pregnancy in **PKU** in your country/region? *

- Yes
- No
- Unsure

14. Do patients/caregivers have to make any payment (full/partial) towards the cost of protein substitutes/medical foods for **PKU** in your country/region? **(tick all that apply)** *

- [ ] Fully paid by government/insurance
- [ ] Fully paid by government/insurance but only under certain conditions e.g. adult full time education, adult unemployment
- [ ] Partly subsidised by government/ insurance, patient pays for some
- [ ] Patient has to pay for all of it
- [ ] Unsure
- [ ] Other

15. Please tell us more about the payment process in your country/region and any costs (e.g. for protein substitutes/medical foods) for your patients with **PKU** (if you do not know, please put UNSURE): *

16. Do **new** protein substitutes/medical foods for **PKU** need to go through a national approval process before they can be given to patients in your country/region? *

- Yes
- No
- Unsure

17. Please tell us more about the approval process for protein substitutes/medical foods for your patients with **PKU** in your country/region (if you do not know, please put UNSURE): *

18. Do you have access to CGMP based protein substitutes/medical foods for **PKU** in your centre/hospital?
*

- ◯ Yes
- ◯ No
- ◯ We have had in the past but not now
- ◯ We will have soon
- ◯ Unsure

19. If you do not have access to CGMP based protein substitutes/medical foods for **PKU**, what is the reason for this? **(tick all that apply)** *

- [ ] Too expensive
- [ ] Lack of evidence for clinical efficacy
- [ ] Not approved for use by government/insurance system
- [ ] Not aware of their availability
- [ ] Not been promoted by manufacturers/wholesalers
- [ ] Not included in national guidelines
- [ ] Not included in international guidelines
- [ ] Unsure
- [ ] Other

20. Do patients in your country/region ever experience supply interruption when taking CGMP based protein substitutes/medical foods for **PKU**? *

- ◯ Yes
- ◯ No
- ◯ Unsure
- ◯ CGMP not available

21. If your patients do experience supply interruption when taking CGMP based protein substitutes/medical foods for **PKU**, please explain why. *

22. Are CGMP based protein substitutes/medical foods for **PKU** that are available in your country generally more expensive than amino acid based protein substitutes? *

- ◯ Yes
- ◯ No
- ◯ Unsure
- ◯ CGMP not available

23. Do you have any further comments about access to CGMP based protein substitutes/medical foods for **PKU**? (e.g. those responsible for choosing products, whether they are approved for use for all patient groups, why they may not be available)

Clinical practice with CGMP based protein substitutes/medical foods

24. What if any, do you think are the **clinical advantages** of CGMP based protein substitutes/medical foods for **PKU** compared to amino acid based protein substitutes/medical foods? (**choose up to 5 options you consider to be the most important**) *

Please select at most 5 options.

- ☐ Improved taste/palatability
- ☐ Improved satiety
- ☐ Improved nitrogen retention
- ☐ Reduced blood phenylalanine variability
- ☐ Improved body composition
- ☐ Improved growth
- ☐ Improved bone density
- ☐ They have prebiotic effects
- ☐ Improved gastrointestinal symptoms e.g. less stomach ache, reflux
- ☐ They have antimicrobial effects
- ☐ They have immunomodulatory action

- [ ] Rich in large neutral amino acids (LNAA)
- [ ] Improved blood tyrosine
- [ ] Improved breath odour
- [ ] Improved absorption
- [ ] Better for dental health
- [ ] Better renal function
- [ ] Better for oxidative stress
- [ ] They alleviate symptoms of nausea/ morning sickness in pregnancy
- [ ] Other

25. What if any, do you think are the **clinical disadvantages** of CGMP based protein substitutes/medical foods for **PKU** compared to amino acid based protein substitutes/ medical foods? (**choose up to 5 options you consider to be the most important**) *

Please select at most 5 options.

- ☐ Limited long term data to support their efficacy
- ☐ Evidence for clinical benefits are based mostly on non-PKU studies or animal studies
- ☐ Contains residual phenylalanine
- ☐ Amino acid profile not ideal
- ☐ Worse taste/palatability
- ☐ Poor absorption
- ☐ Limited efficacy data in pregnancy/lactation
- ☐ Limited evidence to support children aged 3 years or less
- ☐ Some products too high in calories
- ☐ Other

26. Do you consider that the residual phenylalanine in CGMP based protein substitutes/medical foods negatively affects blood phenylalanine control in: (please put not applicable if you do not care for any of these categories of patient) *

	Yes	No	Sometimes
Children aged ≤12 years	○	○	○
Teenagers aged 13-18 years	○	○	○
Adults >18 years	○	○	○
Pregnancy 1st trimester	○	○	○
Pregnancy 2nd trimester	○	○	○
Pregnancy 3rd trimester	○	○	○

27. What percentage of your patients with **PKU** are currently taking at least some CGMP based protein substitutes/medical foods? *

- None
- ≤ 5%
- ≤ 10%
- ≤ 25%
- ≤ 50%
- ≤ 75%
- ≤ 100%
- CGMP not available

28. Approximately how many of your patients with **PKU** aged <1 year are **currently** taking at least some CGMP based protein substitutes/medical foods? (if you do not care for patients in this age group please put N/A, if you do not know please put UNSURE) *

29. Approximately how many of your patients with **PKU** aged 1-4 years are **currently** taking at least some CGMP based protein substitutes/medical foods? (if you do not care for patients in this age group please put N/A, if you do not know please put UNSURE) *

30. Approximately how many of your patients with **PKU** aged 5-12 years are **currently** taking at least some CGMP based protein substitutes/medical foods? (if you do not care for patients in this age group please put N/A, if you do not know please put UNSURE) *

31. Approximately how many of your patients with **PKU** aged 13-18 years are **currently** taking at least some CGMP based protein substitutes/medical foods? (if you do not care for patients in this age group please put N/A, if you do not know please put UNSURE) *

32. Approximately how many of your patients with **PKU** aged >18 years are **currently** taking at least some CGMP based protein substitutes/medical foods? (if you do not care for patients in this age group please put N/A, if you do not know please put UNSURE) *

33. Approximately how many of your late treated patients with **PKU** are **currently** taking at least some CGMP based protein substitutes/medical foods? (if you do not care for any late treated patients please put N/A, if you do not know please put UNSURE) *

34. Approximately how many of your pregnant patients with **PKU** (**past or current**) have taken/are taking at least some CGMP based protein substitutes/medical foods? (if you do not care for patients who are pregnant please put N/A, if you do not know please put UNSURE) *

35. Approximately how many of your lactating patients with **PKU** (**past or current**) have taken/are taking at least some CGMP based protein substitutes/medical foods? (if you do not care for patients who are lactating please put N/A, if you do not know please put UNSURE) *

36. How many doses a day do you usually recommend from CGMP based protein substitutes/medical foods for your patients with **PKU (tick all that apply)**? *

- [ ] 1 dose
- [ ] 2 doses
- [ ] 3 doses
- [ ] 4 doses
- [ ] It varies depending on the patient preference
- [ ] It varies according to the patients' blood phenylalanine level

37. Would you/ do you use CGMP based protein substitutes/medical foods as your **first choice** protein substitute/medical food for **PKU** in any of the following clinical areas? (**tick all that apply**) *

- [ ] Poor adherence with amino acid based protein substitutes
- [ ] Poor metabolic control
- [ ] Good metabolic control
- [ ] Refusal to take amino acid based protein substitutes
- [ ] Infants (up to 1 year of age)
- [ ] Children (1-12 years)
- [ ] Adolescents (13-18 years)
- [ ] Adults (>18 years)
- [ ] Patients recommencing/starting dietary treatment in adult life
- [ ] Unable to drink 'juice' amino acid based protein substitutes
- [ ] On adjunct drug treatment e.g. sapropterin
- [ ] Pregnancy (e.g. due to nausea/vomiting on amino acid based protein substitutes)
- [ ] I never use CGMP based protein substitutes/medical foods as my first choice
- [ ] Other

38. In your clinical practice with the use of CGMP in patients with **PKU,** which of the following have you observed? **(choose up to 5)** *

Please select at most 5 options.

- [ ] Do not use CGMP
- [ ] No clinical advantage observed
- [ ] Some patients report better palatability
- [ ] Better adherence with protein substitute for some patients
- [ ] Enabled some patients to restart dietary management
- [ ] Some patients report improved satiety
- [ ] Reduced blood phenylalanine variability
- [ ] Improved patient body composition
- [ ] Improved growth in some patients
- [ ] Increased overweight/obesity
- [ ] Less gastrointestinal symptoms e.g. less stomach ache, reflux
- [ ] Higher blood phenylalanine levels
- [ ] Improved blood tyrosine
- [ ] Less breath odour
- [ ] Better renal function
- [ ] Alleviated symptoms of nausea/ morning sickness in pregnancy

☐ Other

39. Do you have any further comments about your clinical practice with CGMP based protein substitutes/medical foods for **PKU**? (e.g. issues with residual phenylalanine, percentage of patients taking CGMP products, number of doses of CGMP prescribed, reasons for choice of CGMP products and any observed benefits or disadvantages)

Nutritional profile of CGMP based protein substitutes/medical foods

40. Are you satisfied with the current phenylalanine content of most of the CGMP based protein substitutes/medical foods available for **PKU**? *

- Too high
- Too low
- Just right
- Unsure

41. Are you satisfied with the current tyrosine content of most of the CGMP based protein substitutes/medical foods available for **PKU**? *

- Too high
- Too low
- Just right
- Unsure

42. Would you/ do you change the dietary phenylalanine prescription for **PKU** according to the phenylalanine content of the CGMP protein substitute/medical food? *

- ◯ Yes
- ◯ No
- ◯ Sometimes

43. Are you satisfied with the overall amino acid composition of most of the current CGMP based protein substitutes/medical foods available for **PKU**? *

- ◯ Yes
- ◯ No
- ◯ Unsure

44. What modifications do you think are needed to the amino acid profile of CGMP protein substitutes/medical foods for patients with **PKU**?

45. Are you satisfied with the overall energy content of most of the current CGMP based protein substitutes/medical foods available for **PKU**? *

- Yes
- No
- Unsure

46. What modifications do you think are needed to the energy content of CGMP protein substitutes/medical foods for patients with **PKU**?

47. Do you have other concerns about the nutritional quality of current CGMP products for **PKU**? (**tick all that apply**) *

- ☐ Some are too high in carbohydrate
- ☐ Some are too high in sugar
- ☐ Some are too high in fat
- ☐ Some are too low in fat
- ☐ Some do not contain added long chain fatty acids
- ☐ Some are too high in vitamin and minerals
- ☐ Some are too low in vitamin and minerals
- ☐ The osmolality is too high in some
- ☐ No other concerns
- ☐ Unsure
- ☐ Other

48. If you have concerns about the vitamin and mineral content of some/all CGMP based protein substitutes/medical foods for **PKU**, which vitamins and minerals in particular concern you?

49. Do you think all CGMP based protein substitutes/medical foods for **PKU** should contain vitamins and minerals? *

- Yes
- No
- Unsure

50. If you do not think all CGMP based protein substitutes/medical foods should contain vitamins and minerals for **PKU** what is your reason?

51. Do you have any further comments about the nutritional profile of CGMP based protein substitutes/medical foods for **PKU**? (e.g. phenylalanine, tyrosine, energy, carbohydrate, sugar or fat content)

Presentation of CGMP based protein substitutes/medical foods

52. How many different powdered CGMP protein substitutes/medical foods are available for your patients with **PKU** (do not count different flavours or different protein equivalents of the same product)? *

- None
- 1
- 2-5
- 6-10
- > 10
- Unsure

53. How many different liquid CGMP protein substitutes/medical foods are available for your patients with **PKU** (do not count different flavours or different protein equivalents of the same product)? *

- ◯ None
- ◯ 1
- ◯ 2-5
- ◯ 6-10
- ◯ > 10
- ◯ Unsure

54. How many different CGMP protein substitute/medical food bars are available for your patients with **PKU** (do not count different flavours or different protein equivalents of the same product)? *

- ◯ None
- ◯ 1
- ◯ 2-5
- ◯ 6-10
- ◯ > 10
- ◯ Unsure

55. Do you have a preference for the format of CGMP protein substitutes/medical foods for your patients with **PKU**? **(tick all that apply)** *

- ☐ Liquid
- ☐ Powder
- ☐ Bars
- ☐ No preference
- ☐ Unsure

56. Do you think the flavour options for CGMP protein substitutes/medical foods are adequate for your patients with **PKU**? *

- ◯ Yes
- ◯ No
- ◯ Unsure
- ◯ CGMP not available

57. Do you have any further comments about the presentation of CGMP based protein substitutes/medical foods for **PKU**?

Marketing of CGMP based protein substitutes/medical foods

58. Please choose your clinical preference from the following protein substitutes/medical foods for **PKU**? *

- ○ Amino acid based
- ○ CGMP based
- ○ Slow release amino acids
- ○ A combination of CGMP and amino acid based
- ○ A combination of CGMP and slow release amino acids
- ○ A combination of amino acid based and slow release amino acids
- ○ Unsure
- ○ Other

59. Please explain the reasons for your previous answer *

60. Do you have any further comments about CGMP based protein substitutes/medical foods for **PKU**?

TYROSINAEMIA (TYR)

61. Do you care for patients with **tyrosinaemia (TYR) (Type I, II or III)** on dietary treatment? *

- ◯ Yes
- ◯ No

62. Do you normally care for paediatric or adult patients with **TYR**, or both? *

- ◯ Paediatric (0-18 years)
- ◯ Adult (>18 years)
- ◯ Both paediatric and adult

63. Approximately how many patients with **TYR** on dietary treatment do you care for? *

- ◯ <10
- ◯ 10-20
- ◯ 21-50
- ◯ 51-100
- ◯ 101-150
- ◯ >150

Access to CGMP based protein substitutes/medical foods (TYR)

64. Who is responsible for choosing the type of protein substitute/medical foods that you use in your hospital/centre for **TYR (tick all that apply)**? *

- [ ] Dietitian/nutritionist
- [ ] Doctor/physician
- [ ] Multidisciplinary team
- [ ] Patients/carers
- [ ] Joint decision between patient/carer and dietitian/nutritionist
- [ ] Joint decision between patient/carer and doctor/physician
- [ ] Other

65. Are CGMP based protein substitutes/medical foods approved for use in **TYR** by regulatory processes in your country/region/state? *

- Yes
- No
- They are in the process of being approved
- Unsure

66. Comments

67. Is there a minimum age for use of CGMP based protein substitutes/medical foods for use in **TYR** in your country/region? *

- Yes
- No
- Unsure
- CGMP not available

68. If yes, from what age can CGMP based protein substitutes be used for **TYR**? *

69. Are CGMP based protein substitutes/medical foods approved for use in pregnancy in **TYR** in your country/region? *

- Yes
- No
- Unsure

70. Do patients/caregivers have to make any payment (full/partial) towards the cost of protein substitutes/medical foods for **TYR** in your country/region? **(tick all that apply)** *

- ☐ Fully paid by government/insurance
- ☐ Fully paid by government/insurance but only under certain conditions e.g. adult full time education, adult unemployment
- ☐ Partly subsidised by government/ insurance, patient pays for some
- ☐ Patient has to pay for all of it
- ☐ Unsure
- ☐ Other

71. Please tell us more about the payment process in your country/region and any costs (e.g. for protein substitutes/medical foods) for your patients with **TYR** (if you do not know, please put UNSURE): *

72. Do **new** protein substitutes/medical foods for **TYR** need to go through a national approval process before they can be given to patients in your country/region? *

- ◯ Yes
- ◯ No
- ◯ Unsure

73. Please tell us more about the approval process for protein substitutes/medical foods for your patients with **TYR** in your country/region (if you do not know, please put UNSURE): *

74. Do you have access to CGMP based protein substitutes/medical foods for **TYR** in your centre/hospital? *

- Yes
- No
- We have had in the past but not now
- We will have soon
- Unsure

75. If you do not have access to CGMP based protein substitutes/medical foods for **TYR**, what is the reason for this? **(tick all that apply)** *

- [ ] Too expensive
- [ ] Lack of evidence for clinical efficacy
- [ ] Not approved for use by government/insurance system
- [ ] Not aware of their availability
- [ ] Not been promoted by manufacturers/wholesalers
- [ ] Not included in national guidelines
- [ ] Not included in international guidelines
- [ ] Unsure
- [ ] Other

76. Do patients in your country/region ever experience supply interruption when taking CGMP based protein substitutes/medical foods for **TYR**? *

- ◯ Yes
- ◯ No
- ◯ Unsure
- ◯ CGMP not available

77. If your patients do experience supply interruption when taking CGMP based protein substitutes/medical foods for **TYR**, please explain why. *

78. Are CGMP based protein substitutes/medical foods for **TYR** that are available in your country generally more expensive than amino acid based protein substitutes? *

- Yes
- No
- Unsure
- CGMP not available

79. Do you have any further comments about access to CGMP based protein substitutes/medical foods for **TYR**? (e.g. those responsible for choosing products, whether they are approved for use for all patient groups, why they may not be available)

Clinical practice with CGMP based protein substitutes/medical foods (TYR)

80. What if any, do you think are the **clinical advantages** of CGMP based protein substitutes/medical foods for **TYR** compared to amino acid based protein substitutes/medical foods? (**choose up to 5 options you consider to be the most important**) *

Please select at most 5 options.

- [ ] Improved taste/palatability
- [ ] Improved satiety
- [ ] Improved nitrogen retention
- [ ] Reduced blood tyrosine variability
- [ ] Improved body composition
- [ ] Improved growth
- [ ] Improved bone density
- [ ] They have prebiotic effects
- [ ] Improved gastrointestinal symptoms e.g. less stomach ache, reflux
- [ ] They have antimicrobial effects
- [ ] They have immunomodulatory action

- [ ] Rich in LNAA
- [ ] Improved blood phenylalanine
- [ ] Improved breath odour
- [ ] Improved absorption
- [ ] Better for dental health
- [ ] Better renal function
- [ ] Better for oxidative stress
- [ ] They alleviate symptoms of nausea/ morning sickness in pregnancy
- [ ] Other

81. What if any, do you think are the **clinical disadvantages** of CGMP based protein substitutes/medical foods for **TYR** compared to amino acid based protein substitutes/ medical foods? (**choose up to 5 options you consider to be the most important**) *

Please select at most 5 options.

- [ ] Limited long term data to support their efficacy
- [ ] Evidence for clinical benefits are based mostly on non-TYR studies or animal studies
- [ ] Contains residual tyrosine
- [ ] Amino acid profile not ideal
- [ ] Worse taste/palatability
- [ ] Poor absorption
- [ ] Limited efficacy data in pregnancy/lactation
- [ ] Limited evidence to support children aged 3 years or less
- [ ] Some products too high in calories
- [ ] Other

82. Do you consider that the residual tyrosine and phenylalanine in CGMP based protein substitutes/medical foods negatively affects blood tyrosine in: (please put not applicable if you do not care for any of these categories of patient)
*

	Yes	No	Sometimes
Children aged ≤12 years	○	○	○
Teenagers aged 13-18 years	○	○	○
Adults >18 years	○	○	○
Pregnancy 1st trimester	○	○	○
Pregnancy 2nd trimester	○	○	○
Pregnancy 3rd trimester	○	○	○

83. What percentage of your patients with **TYR** are currently taking CGMP based protein substitutes/medical foods? *

- None
- ≤ 5%
- ≤ 10%
- ≤ 25%
- ≤ 50%
- ≤ 75%
- ≤ 100%
- CGMP not available

84. Approximately how many of your patients with **TYR** aged <1 year are **currently** taking at least some CGMP based protein substitutes/medical foods? (if you do not care for patients in this age group please put N/A, if you do not know please put UNSURE) *

85. Approximately how many of your patients with **TYR** aged 1-4 years are **currently** taking at least some CGMP based protein substitutes/medical foods? (if you do not care for patients in this age group please put N/A, if you do not know please put UNSURE) *

86. Approximately how many of your patients with **TYR** aged 5-12 years are **currently** taking at least some CGMP based protein substitutes/medical foods? (if you do not care for patients in this age group please put N/A, if you do not know please put UNSURE) *

87. Approximately how many of your patients with **TYR** aged 13-18 years are **currently** taking at least some CGMP based protein substitutes/medical foods? (if you do not care for patients in this age group please put N/A, if you do not know please put UNSURE) *

88. Approximately how many of your patients with **TYR** aged >18 years are **currently** taking at least some CGMP based protein substitutes/medical foods? (if you do not care for patients in this age group please put N/A, if you do not know please put UNSURE) *

89. Approximately how many of your pregnant patients with **TYR** (**past or current**) have taken/are taking at least some CGMP based protein substitutes/medical foods? (if you do not care for patients who are pregnant please put N/A, if you do not know please put UNSURE) *

90. Approximately how many of your lactating patients with **TYR** (**past or current**) have taken/are taking at least some CGMP based protein substitutes/medical foods? (if you do not care for patients who are lactating please put N/A, if you do not know please put UNSURE) *

91. How many doses a day do you usually recommend from CGMP based protein substitutes/medical foods for your patients with **TYR (tick all that apply)**? *

- [ ] 1 dose
- [ ] 2 doses
- [ ] 3 doses
- [ ] 4 doses
- [ ] It varies depending on the patient preference
- [ ] It varies according to the patients' blood tyrosine level

92. Would you/ do you use CGMP based protein substitutes/medical foods as your **first choice** protein substitute/medical food for **TYR** in any of the following clinical areas? (**tick all that apply**) *

- [ ] Poor adherence with amino acid based protein substitutes
- [ ] Poor metabolic control
- [ ] Good metabolic control
- [ ] Refusal to take amino acid based protein substitutes
- [ ] Infants (up to 1 year of age)
- [ ] Children (1-12 years)
- [ ] Adolescents (13-18 years)
- [ ] Adults (>18 years)
- [ ] Patients recommencing/starting dietary treatment in adult life
- [ ] Unable to drink 'juice' amino acid based protein substitutes
- [ ] On adjunct drug treatment
- [ ] Pregnancy (e.g. due to nausea/vomiting on amino acid based protein substitutes)
- [ ] I never use CGMP based protein substitutes/medical foods as my first choice
- [ ] Other

93. In your clinical practice with the use of CGMP in patients with **TYR,** which of the following have you observed? **(choose up to 5)** *

Please select at most 5 options.

- [ ] Do not use CGMP
- [ ] No clinical advantage observed
- [ ] Some patients report better palatability
- [ ] Better adherence with protein substitute for some patients
- [ ] Enabled some patients to restart dietary management
- [ ] Some patients report improved satiety
- [ ] Reduced blood tyrosine variability
- [ ] Improved patient body composition
- [ ] Improved growth
- [ ] Increased overweight/obesity
- [ ] Less gastrointestinal symptoms e.g. less stomach ache, reflux
- [ ] Higher blood tyrosine levels
- [ ] Improved blood phenylalanine
- [ ] Less breath odour
- [ ] Better renal function
- [ ] Alleviated symptoms of nausea/ morning sickness in pregnancy

☐ Other

94. Do you have any further comments about your clinical practice with CGMP based protein substitutes/medical foods for **TYR**? (e.g. issues with residual phenylalanine and/or tyrosine, percentage of patients taking CGMP products, number of doses of CGMP prescribed, reasons for choice of CGMP products and any observed benefits or disadvantages)

Nutritional profile of CGMP based protein substitutes/medical foods (TYR)

95. Are you satisfied with the current tyrosine content of most of the CGMP based protein substitutes/medical foods available for **TYR**? *

- Too high
- Too low
- Just right
- Unsure

96. Are you satisfied with the current phenylalanine content of most of the CGMP based protein substitutes/medical foods available for **TYR**? *

- Too high
- Too low
- Just right
- Unsure

97. Would you/ do you change the dietary tyrosine/phenylalanine prescription for **TYR** according to the tyrosine/phenylalanine content of the CGMP protein substitute/medical food? *

- Yes
- No
- Sometimes

98. Are you satisfied with the overall amino acid composition of most of the current CGMP based protein substitutes/medical foods available for **TYR**? *

- Yes
- No
- Unsure

99. What modifications do you think are needed to the amino acid profile of CGMP protein substitutes/medical foods for patients with **TYR**?

100. Are you satisfied with the overall energy content of most of the current CGMP based protein substitutes/medical foods available for **TYR**? *

- ◯ Yes
- ◯ No
- ◯ Unsure

101. What modifications do you think are needed to the energy content of CGMP protein substitutes/medical foods for patients with **TYR**?

102. Do you have other concerns about the nutritional quality of current CGMP products for **TYR**? (**tick all that apply**) *

- [ ] Some are too high in carbohydrate
- [ ] Some are too high in sugar
- [ ] Some are too high in fat
- [ ] Some are too low in fat
- [ ] Some do not contain added long chain fatty acids
- [ ] Some are too high in vitamin and minerals
- [ ] Some are too low in vitamin and minerals
- [ ] The osmolality is too high in some
- [ ] No other concerns
- [ ] Unsure
- [ ] Other

103. If you have concerns about the vitamin and mineral content of some/all CGMP based protein substitutes/medical foods for **TYR**, which vitamins and minerals in particular concern you?

104. Do you think all CGMP based protein substitutes/medical foods for **TYR** should contain vitamins and minerals? *

- Yes
- No
- Unsure

105. If you do not think all CGMP based protein substitutes/medical foods should contain vitamins and minerals for **TYR** what is your reason?

106. Do you have any further comments about the nutritional profile of CGMP based protein substitutes/medical foods for **TYR**? (e.g. phenylalanine, tyrosine, energy, carbohydrate, sugar or fat content)

Presentation of CGMP based protein substitutes/medical foods (TYR)

107. How many different powdered CGMP protein substitutes/medical foods are available for your patients with **TYR** (do not count different flavours or different protein equivalents of the same product)? *

- ○ None
- ○ 1
- ○ 2-5
- ○ Unsure

108. How many different liquid CGMP protein substitutes/medical foods are available for your patients with **TYR** (do not count different flavours or different protein equivalents of the same product)? *

- ○ None
- ○ 1
- ○ 2-5
- ○ Unsure

109. How many different CGMP protein substitute/medical food bars are available for your patients with **TYR** (do not count different flavours or different protein equivalents of the same product)? *

- ○ None
- ○ 1
- ○ 2-5
- ○ Unsure

110. Do you have a preference for the format of CGMP protein substitutes/medical foods for your patients with **TYR**? **(tick all that apply)** *

- ☐ Liquid
- ☐ Powder
- ☐ Bars
- ☐ No preference
- ☐ Unsure

111. Do you think the flavour options for CGMP protein substitutes/medical foods are adequate for your patients with **TYR**? *

- ◯ Yes
- ◯ No
- ◯ Unsure
- ◯ CGMP not available

112. Do you have any further comments about the presentation of CGMP based protein substitutes/medical foods for **TYR**?

Marketing of CGMP based protein substitutes/medical foods (TYR)

113. Please choose your clinical preference from the following protein substitutes/medical foods for **TYR**? *

- ◯ Amino acid based
- ◯ CGMP based
- ◯ A combination of CGMP and amino acid based
- ◯ Unsure
- ◯ Other

114. Please explain the reasons for your previous answer *

115. Do you have any further comments about CGMP based protein substitutes/medical foods for **TYR**?

ALKAPTONURIA (ALK)

116. Do you care for patients with **alkaptonuria (ALK)** on dietary treatment? *

- ◯ Yes
- ◯ No

117. Do you normally care for paediatric or adult patients with **ALK**, or both? *

- ◯ Paediatric (0-18 years)
- ◯ Adult (>18 years)
- ◯ Both paediatric and adult

118. Approximately how many patients with **ALK** on dietary treatment do you care for? *

- ◯ <10
- ◯ 10-20
- ◯ 21-50
- ◯ 51-100
- ◯ 101-150
- ◯ >150

Access to CGMP based protein substitutes/medical foods (ALK)

119. Who is responsible for choosing the type of protein substitute/medical foods that you use in your hospital/centre for **ALK (tick all that apply)**? *

- [ ] Dietitian/nutritionist
- [ ] Doctor/physician
- [ ] Multidisciplinary team
- [ ] Patients/carers
- [ ] Joint decision between patient/carer and dietitian/nutritionist
- [ ] Joint decision between patient/carer and doctor/physician
- [ ] Other

120. Are CGMP based protein substitutes/medical foods approved for use in **ALK** by regulatory processes in your country/region/state? *

- Yes
- No
- They are in the process of being approved
- Unsure

121. Comments

122. Is there a minimum age for use of CGMP based protein substitutes/medical foods for use in **ALK** in your country/region? *

- Yes
- No
- Unsure
- CGMP not available

123. If yes, from what age can CGMP based protein substitutes be used for **ALK**? *

124. Are CGMP based protein substitutes/medical foods approved for use in pregnancy in **ALK** in your country/region? *

- Yes
- No
- Unsure

125. Do patients/caregivers have to make any payment (full/partial) towards the cost of protein substitutes/medical foods for **ALK** in your country/region? **(tick all that apply)** *

- ☐ Fully paid by government/insurance
- ☐ Fully paid by government/insurance but only under certain conditions e.g. adult full time education, adult unemployment
- ☐ Partly subsidised by government/ insurance, patient pays for some
- ☐ Patient has to pay for all of it
- ☐ Unsure
- ☐ Other

126. Please tell us more about the payment process in your country/region and any costs (e.g. for protein substitutes/medical foods) for your patients with **ALK** (if you do not know, please put UNSURE): *

127. Do **new** protein substitutes/medical foods for **ALK** need to go through a national approval process before they can be given to patients in your country/region? *

- ◯ Yes
- ◯ No
- ◯ Unsure

128. Please tell us more about the approval process for protein substitutes/medical foods for your patients with **ALK** in your country/region (if you do not know, please put UNSURE): *

129. Do you have access to CGMP based protein substitutes/medical foods for **ALK** in your centre/hospital?
*

- ◯ Yes
- ◯ No
- ◯ We have had in the past but not now
- ◯ We will have soon
- ◯ Unsure

130. If you do not have access to CGMP based protein substitutes/medical foods for **ALK**, what is the reason for this? **(tick all that apply)** *

- [ ] Too expensive
- [ ] Lack of evidence for clinical efficacy
- [ ] Not approved for use by government/insurance system
- [ ] Not aware of their availability
- [ ] Not been promoted by manufacturers/wholesalers
- [ ] Not included in national guidelines
- [ ] Not included in international guidelines
- [ ] Unsure
- [ ] Other

131. Do patients in your country/region ever experience supply interruption when taking CGMP based protein substitutes/medical foods for **ALK**? *

- Yes
- No
- Unsure
- CGMP not available

132. If your patients do experience supply interruption when taking CGMP based protein substitutes/medical foods for **ALK**, please explain why. *

133. Are CGMP based protein substitutes/medical foods for **ALK** that are available in your country generally more expensive than amino acid based protein substitutes?
*

- Yes
- No
- Unsure
- CGMP not available

134. Do you have any further comments about access to CGMP based protein substitutes/medical foods for **ALK**? (e.g. those responsible for choosing products, whether they are approved for use for all patient groups, why they may not be available)

Clinical practice with CGMP based protein substitutes/medical foods (ALK)

135. What if any, do you think are the **clinical advantages** of CGMP based protein substitutes/medical foods for **ALK** compared to amino acid based protein substitutes/medical foods? (**choose up to 5 options you consider to be the most important**) *

Please select at most 5 options.

- [ ] Improved taste/palatability
- [ ] Improved satiety
- [ ] Improved nitrogen retention
- [ ] Reduced blood tyrosine variability
- [ ] Improved body composition
- [ ] Improved growth
- [ ] Improved bone density
- [ ] They have prebiotic effects
- [ ] Improved gastrointestinal symptoms e.g. less stomach ache, reflux
- [ ] They have antimicrobial effects
- [ ] They have immunomodulatory action

- [ ] Rich in LNAA
- [ ] Improved blood phenylalanine
- [ ] Improved breath odour
- [ ] Improved absorption
- [ ] Better for dental health
- [ ] Better renal function
- [ ] Better for oxidative stress
- [ ] They alleviate symptoms of nausea/ morning sickness in pregnancy
- [ ] Other

136. What if any, do you think are the **clinical disadvantages** of CGMP based protein substitutes/medical foods for **ALK** compared to amino acid based protein substitutes/ medical foods? (**choose up to 5 options you consider to be the most important**) *

Please select at most 5 options.

- [ ] Limited long term data to support their efficacy
- [ ] Evidence for clinical benefits are based mostly on non-ALK studies or animal studies
- [ ] Contains residual tyrosine
- [ ] Amino acid profile not ideal
- [ ] Worse taste/palatability
- [ ] Poor absorption
- [ ] Limited efficacy data in pregnancy/lactation
- [ ] Some products too high in calories
- [ ] Other

137. Do you consider that the residual tyrosine and phenylalanine in CGMP based protein substitutes/medical foods negatively affects blood tyrosine in: (please put not applicable if you do not care for any of these categories of patient)
*

	Yes	No	Sometimes
Children aged ≤12 years	○	○	○
Teenagers aged 13-18 years	○	○	○
Adults >18 years	○	○	○
Pregnancy 1st trimester	○	○	○
Pregnancy 2nd trimester	○	○	○
Pregnancy 3rd trimester	○	○	○

138. What percentage of your patients with **ALK** are currently taking at least some CGMP based protein substitutes/medical foods? *

- None
- ≤ 5%
- ≤ 10%
- ≤ 25%
- ≤ 50%
- ≤ 75%
- ≤ 100%
- CGMP not available

139. Approximately how many of your patients with **ALK** aged 11-18 years are **currently** taking at least some CGMP based protein substitutes/medical foods? (if you do not care for patients in this age group please put N/A, if you do not know please put UNSURE) *

140. Approximately how many of your patients with **ALK** aged >18 years are **currently** taking at least some CGMP based protein substitutes/medical foods? (if you do not care for patients in this age group please put N/A, if you do not know please put UNSURE) *

141. How many doses a day do you usually recommend from CGMP based protein substitutes/medical foods for your patients with **ALK (tick all that apply)**? *

- [ ] 1 dose
- [ ] 2 doses
- [ ] 3 doses
- [ ] 4 doses
- [ ] It varies depending on the patient preference
- [ ] It varies according to the patients' blood tyrosine level

142. Would you/ do you use CGMP based protein substitutes/medical foods as your **first choice** protein substitute/medical food for **ALK** in any of the following clinical areas? (**tick all that apply**) *

- ☐ Poor adherence with amino acid based protein substitutes
- ☐ Poor metabolic control
- ☐ Good metabolic control
- ☐ Refusal to take amino acid based protein substitutes
- ☐ Adolescents (13-18 years)
- ☐ Adults (>18 years)
- ☐ Patients recommencing/starting dietary treatment in adult life
- ☐ Unable to drink 'juice' amino acid based protein substitutes
- ☐ On adjunct drug treatment
- ☐ Pregnancy (e.g. due to nausea/vomiting on amino acid based protein substitutes)
- ☐ I never use CGMP based protein substitutes/medical foods as my first choice
- ☐ Other

143. In your clinical practice with the use of CGMP in patients with **ALK,** which of the following have you observed? **(choose up to 5)** *

Please select at most 5 options.

- [ ] Do not use CGMP
- [ ] No clinical advantage observed
- [ ] Some patients report better palatability
- [ ] Better adherence with protein substitute for some patients
- [ ] Enabled some patients to restart dietary management
- [ ] Some Patients report improved satiety
- [ ] Reduced blood tyrosine variability
- [ ] Improved patient body composition
- [ ] Improved growth
- [ ] Increased overweight/obesity
- [ ] Less gastrointestinal symptoms e.g. less stomach ache, reflux
- [ ] Higher blood tyrosine levels
- [ ] Improved blood phenylalanine
- [ ] Less breath odour
- [ ] Better renal function
- [ ] Alleviated symptoms of nausea/ morning sickness in pregnancy
- [ ] Other

144. Do you have any further comments about your clinical practice with CGMP based protein substitutes/medical foods for **ALK**? (e.g. issues with residual phenylalanine and/or tyrosine, percentage of patients taking CGMP products, number of doses of CGMP prescribed, reasons for choice of CGMP products and any observed benefits or disadvantages)

Nutritional profile of CGMP based protein substitutes/medical foods (ALK)

145. Are you satisfied with the current tyrosine content of most of the CGMP based protein substitutes/medical foods available for **ALK**? *

- Too high
- Too low
- Just right
- Unsure

146. Are you satisfied with the current phenylalanine content of most of the CGMP based protein substitutes/medical foods available for **ALK**? *

- Too high
- Too low
- Just right
- Unsure

147. Would you/ do you change the dietary tyrosine/phenylalanine prescription for **ALK** according to the tyrosine/phenylalanine content of the CGMP protein substitute/medical food? *

- Yes
- No
- Sometimes

148. Are you satisfied with the overall amino acid composition of most of the current CGMP based protein substitutes/medical foods available for **ALK**? *

- Yes
- No
- Unsure

149. What modifications do you think are needed to the amino acid profile of CGMP protein substitutes/medical foods for patients with **ALK**?

150. Are you satisfied with the overall energy content of most of the current CGMP based protein substitutes/medical foods available for **ALK**? *

◯ Yes

◯ No

◯ Unsure

151. What modifications do you think are needed to the energy content of CGMP protein substitutes/medical foods for patients with **ALK**?

152. Do you have other concerns about the nutritional quality of current CGMP products for **ALK**? (**tick all that apply**) *

- [ ] Some are too high in carbohydrate
- [ ] Some are too high in sugar
- [ ] Some are too high in fat
- [ ] Some are too low in fat
- [ ] Some do not contain added long chain fatty acids
- [ ] Some are too high in vitamin and minerals
- [ ] Some are too low in vitamin and minerals
- [ ] The osmolality is too high in some
- [ ] No other concerns
- [ ] Unsure
- [ ] Other

153. If you have concerns about the vitamin and mineral content of some/all CGMP based protein substitutes/medical foods for **ALK**, which vitamins and minerals in particular concern you?

154. Do you think all CGMP based protein substitutes/medical foods for **ALK** should contain vitamins and minerals? *

- Yes
- No
- Unsure

155. If you do not think all CGMP based protein substitutes/medical foods should contain vitamins and minerals for **ALK** what is your reason?

156. Do you have any further comments about the nutritional profile of CGMP based protein substitutes/medical foods for **ALK**? (e.g. phenylalanine, tyrosine, energy, carbohydrate, sugar or fat content)

Presentation of CGMP based protein substitutes/medical foods (ALK)

157. How many different powdered CGMP protein substitutes/medical foods are available for your patients with **ALK** (do not count different flavours or different protein equivalents of the same product)? *

- None
- 1
- 2-5
- Unsure

158. How many different liquid CGMP protein substitutes/medical foods are available for your patients with **ALK** (do not count different flavours or different protein equivalents of the same product)? *

- None
- 1
- 2-5
- Unsure

159. How many different CGMP protein substitute/medical food bars are available for your patients with **ALK** (do not count different flavours or different protein equivalents of the same product)? *

- ○ None
- ○ 1
- ○ 2-5
- ○ Unsure

160. Do you have a preference for the format of CGMP protein substitutes/medical foods for your patients with **ALK**? **(tick all that apply)** *

- ☐ Liquid
- ☐ Powder
- ☐ Bars
- ☐ No preference
- ☐ Unsure

161. Do you think the flavour options for CGMP protein substitutes/medical foods are adequate for your patients with **ALK**? *

- ◯ Yes
- ◯ No
- ◯ Unsure
- ◯ CGMP not available

162. Do you have any further comments about the presentation of CGMP protein substitutes/medical foods for **ALK**?

Marketing of CGMP based protein substitutes/medical foods (ALK)

163. Please choose your clinical preference from the following protein substitutes/medical foods for **ALK**? *

- ◯ Amino acid based
- ◯ CGMP based
- ◯ A combination of CGMP and amino acid based
- ◯ Unsure
- ◯ Other

164. Please explain the reasons for your previous answer *

165. Do you have any further comments about CGMP based protein substitutes/medical foods for **ALK**?

This content is neither created nor endorsed by Microsoft. The data you submit will be sent to the form owner.

Microsoft Forms
